# Luteal activity of pregnant rats with hypo-and hyperthyroidism

**DOI:** 10.1186/1757-2215-7-75

**Published:** 2014-07-12

**Authors:** Juneo Freitas Silva, Natália Melo Ocarino, Rogéria Serakides

**Affiliations:** 1 Departamento de Clínica e Cirurgia Veterinária, Escola de Veterinária, Universidade Federal de Minas Gerais, Av. Antônio Carlos, 6627, 31270-901 Belo Horizonte, MG, Brazil

**Keywords:** Hypothyroidism, Hyperthyroidism, Corpus luteum, Pregnancy

## Abstract

**Background:**

Luteal activity is dependent on the interaction of various growth factors, cytokines and hormones, including the thyroid hormones, being that hypo- and hyperthyroidism alter the gestational period and are also a cause of miscarriage and stillbirth. Because of that, we evaluated the proliferation, apoptosis and expression of angiogenic factors and COX-2 in the corpus luteum of hypo- and hyperthyroid pregnant rats.

**Methods:**

Seventy-two adult female rats were equally distributed into three groups: hypothyroid, hyperthyroid and control. Hypo- and hyperthyroidism were induced by the daily administration of propylthiouracil and L-thyroxine, respectively. The administration began five days before becoming pregnant and the animals were sacrificed at days 10, 14, and 19 of gestation. We performed an immunohistochemical analysis to evaluate the expression of CDC-47, VEGF, Flk-1 (VEGF receptor) and COX-2. Apoptosis was evaluated by the TUNEL assay. We assessed the gene expression of VEGF, Flk-1, caspase 3, COX-2 and PGF2α receptor using real time RT-PCR. The data were analyzed by SNK test.

**Results:**

Hypothyroidism reduced COX-2 expression on day 10 and 19 (P < 0.05), endothelial/pericyte and luteal cell proliferation on day 10 and 14 (p < 0.05), apoptotic cell numbers on day 19 (p < 0.05) and the expression of Flk-1 and VEGF on day 14 and 19, respectively (p < 0.05). Hyperthyroidism increased the expression of COX-2 on day 19 (P < 0.05) and the proliferative activity of endothelial/pericytes cells on day 14 (p <0.05), as well as the expression of VEGF and Flk-1 on day 19 (P < 0.05).

**Conclusions:**

Hypothyroidism reduces the proliferation, apoptosis and expression of angiogenic factors and COX-2in the corpus luteum of pregnant rats, contrary to what is observed in hyperthyroid animals, being this effect dependent of the gestational period.

## Background

The corpus luteum is a transient, highly regulated endocrine gland which develops from the luteinization of the follicular cells of the granulosa and theca after ovulation. The synthesis of progesterone is the main function of the corpus luteum, which is essential for the establishment and maintenance of pregnancy [1,2]. Thus, luteal dysfunction in women and pregnant animals is associated with the failure of the embryo implantation and abortion [3].

Luteal activity is dependent on the interaction of various growth factors, cytokines and hormones, including the thyroid hormones [4]. The corpus luteum has cellular and molecular mechanisms that are well-coordinated so that its development, maintenance and regression occur correctly [5]. For mechanistic coordination to be achieved, the balance among such varying cellular processes as proliferation, differentiation, cell migration, angiogenesis and apoptosis is critical [6,7]. An example of this coordination occurs during luteinization, during which the cells of the corpus luteum cease their proliferative activity to perform steroidogenesis and to survive in the new luteal environment [8].

If these cellular and molecular mechanisms do not occur in a coordinated manner, early luteal regression may interrupt gestation, with a subsequent miscarriage or premature delivery [9,10]. Hypo- and hyperthyroidism in rats alter the gestational period, causing prolonged gestation and preterm births, respectively [4]. Hypo- and hyperthyroidism in humans are also a cause of miscarriage and stillbirth [11,12]. One hypothesis of this study is that one of the mechanisms by which thyroid dysfunctions affects pregnancy is by altering the proliferative and apoptotic activities of the corpus luteum. The study of the balance between these processes is important and the cell division control protein 47 (CDC47) and caspase 3 have been used as a good marker for cell proliferation and apoptosis, respectively [13,14]. CDC47 is essential for initiation of DNA replication [15], while caspase-3 is required for DNA fragmentation and some of the typical morphological changes of cells undergoing apoptosis [16].

However, angiogenesis is also of fundamental importance for luteal activity during gestation. Pregnant women with luteal hypofunction and low luteal progesterone production have an inadequate blood supply to the corpus luteum [3]. Such a deficiency in luteal angiogenesis has been reported to substantially contribute to subfertility [17], particularly regarding the associated low expression of vascular endothelial growth factor (VEGF) and/or its receptor Flk-1 [18,19]. VEGF is the main factor that regulates angiogenesis by stimulating endothelial cell proliferation and vascular permeability [19]. VEGF also has anti-apoptotic effects [20] and regulates progesterone production by corpus luteum [19]. To date, it is unclear whether the changes in pregnancy induced by hypo- or hyperthyroidism could be due to changes in the expression of VEGF and/or its receptor Flk-1 in the corpus luteum.

The corpus luteum is also under the influence of cyclooxygenase-2 (COX-2), which acts on angiogenesis, steroidogenesis, formation, maintenance and luteal regression through the production of prostaglandin E2 (PGE2) and/or prostaglandin F2α (PGF2α) [21-24]. The PGF2α has luteolytic and vasoactive activities in the corpus luteum [19]. We believe that the changes in the duration of the luteal phases of pregnant animals with hypo- or hyperthyroidism [25-29] can also be a result of changes in COX-2 expression and, consequently, in the expression of prostaglandins.

The objective of this study was to evaluate the proliferation, apoptosis and expression of angiogenic factors and COX-2 in the corpus luteum of pregnant rats with hypo- and hyperthyroidism in different gestational periods.

## Methods

### Induction of thyroid dysfunctions and mating

Seventy-two adult female Wistar rats were housed in a 12-hr light/dark cycle and were fed commercial rat chow and water ad libitum. All procedures were approved by the Institutional Ethics Committee in Animal Experimentation at the Universidade Federal de Minas Gerais (protocol no. 239/2009).

After a 7-day adaptation period, the rats were divided into control, hypothyroid and hyperthyroid groups with 24 animals per group.

Hypothyroidism was induced by the administration of 1 mg/animal/day of propylthiouracil (PTU) (PTU; Sigma-Aldrich, St. Louis, MO, USA) diluted in 5 mL of distilled water, as described by Silva et al. [30], using an orogastric probe. Hyperthyroidism was induced by the administration of 50 μg/animal/day of L-thyroxine (T4) (T2376; Sigma-Aldrich, St. Louis, MO, USA) diluted in 5 mL of distilled water, in accordance with the method of Serakides et al. [31], using an orogastric probe. The rats from the control group received 5 mL of distilled water per day as a placebo.

Five days after treatment initiation, the females were subjected to vaginal cytology to monitor the estrous cycle. Six rats from each group were also euthanized with an overdose of anesthetic for blood collection, measurement of free thyroxine (T4) and confirmation of the induction of thyroid dysfunction. The rats in proestrus were kept in plastic cages with adult male rats for 12 h during the night. After this period, copulation (day 0 of gestation) was confirmed by the presence of spermatozoa in vaginal cytology on the morning afterAnimals in the hypothyroid, hyperthyroid and control groups continued to receive PTU, T4 and water, respectively, throughout the experimental period.

### Hormone analysis

On days 0, 10, 14 and 19 days of gestation, six animals from each group were euthanized by an overdose of anesthetic (2.5% Tionembutal; Abbott, São Paulo, Brazil). At 0 and 19 days of gestation, blood was collected from the rats, and the serum was stored at -20°C for the measurement of free T4, which was performed by the chemiluminescence Elisa technique (sensitivity: 0.4 ng/dl), using commercial kits in accordance with the manufacturer’s instructions (IMMULITE Free T4, Siemens Medical Solutions Diagnostics, Malvern, PA, USA). This assay has been previously used and validated for rat [14,32,33]. The intra- and inter-assay coefficients of variation were 4% and 7%, respectively.

### Necropsy and material collection

At necropsy, the ovaries were collected and dissected. The right ovaries were fixed in 10% neutral and buffered formalin for 24 hours and were processed using a routine paraffin inclusion technique. The corpora lutea of the left ovary were dissected, snap frozen in liquid nitrogen and stored at -80°C to evaluate the gene expression of VEGF, Flk-1, caspase 3, COX-2 and PGF2α receptor using real time RT-PCR. To perform immunohistochemistry and TUNEL assays, histological sections (4 μm) of the right ovary were obtained and placed on silanized slides. All analyses were performed on corpora lutea at 10, 14 and 19 days of gestation.

### Immunohistochemistry

The biotin-streptavidin peroxidase (Streptavidin Peroxidase, Lab Vision Corp., Fremont, CA, USA) technique was used for immunohistochemistry in according to Silva et al. [33]. Antigenic recovery with retrieval solution was performed for 20 minutes. Histological sections were incubated overnight with the primary antibodies anti-VEGF (sc-152, Santa Cruz Biotechnology, CA, USA) (1:100), anti-Flk1 (sc-6251, Santa Cruz Biotechnology, CA, USA) (1:600), anti-COX2 (M3617, Dako, St Louis, MO, USA) (1:50) and anti-CDC47 (47DC141, Neomarkers, Fremont, CA, USA) (1:100). The sections were incubated stepwise for 30 minutes with each of the following solutions: blocking endogenous peroxidase, blocking serum (Ultra Vision Block, Lab Vision Corp., Fremont, CA, USA) and streptavidin peroxidase. Incubation with the secondary antibody (goat biotin, Lab Vision Corp., Fremont, CA, USA) was performed for 45 minutes. The chromogen diaminobenzidine (DAB substrate system, Lab Vision Corp., Fremont, CA, USA) was used for visualization. Sections were counterstained with Harris hematoxylin. A negative control was included by replacing the primary antibodies with IgG.

The number of cells expressing CDC-47 was determined out of a population of 500 cells, and the luteal cells were differentiated from the endothelial cells and pericytes according to their morphological characteristics. Luteal cells were identified by primarily spherical shape, a nucleus containing a prominent nucleolus and the presence of a prominent basement membrane. Vascular cells were identified by association with the vascular compartment, elongated cell and a large nucleus to cytoplasm ratio [34,35]. The corpora lutea were photographed with an Olympus BX-40 microscope and the Spot Color Insight digital camera (SPOTTM, Sterling Heights, Michigan, USA). The number of stained cells was determined using Image Pro Plus® version 4.5 software (Media Cybernetics Manufacturing, Rockville, MD, USA). To ensure the objectivity of the procedure, two images of the stained cells were obtained from each corpora lutea, and counts were taken in three corpora lutea per animal. Data from each corpora lutea were expressed as the percentage of stained cells (%).

The immunostaining intensity and stained area of COX-2, VEGF and Flk-1 in corpora lutea were evaluated. To determine the immunostaining intensity and stained area, images from two fields per CL were photographed with an Olympus BX-40 microscope and the Spot Color Insight digital camera (SPOTTM, Sterling Heights, Michigan, USA), and the immunostaining intensity and stained area were determined using WCIF ImageJ® software (Media Cybernetics Manufacturing, Rockville, MD, USA). Color deconvolution and thresholding of the images were performed. To ensure the objectivity of the procedure, the evaluation was performed in three corpora lutea per animal. Data from each corpus luteum were expressed as the integrated density and stained area in pixels.

### TUNEL assay

Apoptotic cells in the corpora lutea were evaluated by the TUNEL assay using an apoptosis detection kit (TdT-FragEL™ DNA Fragmentation Detection Kit, Calbiochem. San Diego, CA, USA). Antigenic recovery was performed with proteinase K for 20 minutes. The slides were incubated at 37°C with TdT for 1 h and for 30 minutes with each of the following solutions: blocking endogenous peroxidase and streptavidin. The chromogen DAB was utilized and incubated for 15 min. Sections were counterstained with methyl green. A negative control was obtained by replacing TdT with TBS. As a positive control, we employed ovaries with atretic follicles.

The number of apoptotic cells in the corpora lutea was evaluated with an Olympus BX-40 microscope in a 40× objective. Four fields per CL were evaluated, and counts were taken performed in all of the corpora lutea present in the histological sections. Data from each corpora lutea were expressed as the mean number of apoptotic cells/corpus luteum. The data were logarithmically transformed.

### Real time RT-PCR

Total mRNA from corpora lutea was extracted using Trizol reagent (Invitrogen, Carlsbad, CA, USA) and the phenol-chloroform extraction method, according to the manufacturer’s instructions. A total of 1 μg of RNA was used for cDNA synthesis using the SuperScript III Platinum Two-Step qPCR kit with SYBR Green (Invitrogen, Carlsbad, CA, USA). The qRT-PCR reactions were conducted in a Smart Cycler II thermocycler (Cepheid Inc., Sunnyvale, CA, USA). To quantify the cDNA generated by reverse transcription, real-time PCR with SYBR Green I was performed using SYBR Green PCR Master Mix in an Applied Biosystems 7500 Real-Time PCR System (Applied Biosystems, Life Technologies, CA, USA). For negative controls, we used a complete DNA amplification mix in which the target cDNA template was replaced with water. Amplifications were performed using the default cycling conditions: enzyme activation at 95°C for 10 min, 40 cycles of denaturation at 95°C for 15 s, and annealing/extension at 60°C for 60 s. To assess the linearity and efficiency of PCR amplification, standard curves for all transcripts were generated using serial dilutions of cDNA. A melting curve was obtained for the amplification products to ascertain their melting temperatures. The samples were assayed in triplicate and after a gel was run with the reaction product to confirm the gene amplification. The PCR products were separated by electrophoresis on 1% agarose gels and stained with ethidium bromide. Gene expression was calculated using the 2^-∆∆Ct^ method, where the values from the samples were averaged and calibrated in relation to the β-actin CT values. The primers were as follows: forward 5′- GCCCAGACGGGGTGGAGAGT -3′ and reverse 5′- AGGGTTGGCCAGGCTGGGAA -3′ for VEGF (reference sequence: NM_001110336.1; [36]; forward 5′- GTCCGCCGACACTGCTGCAA -3′ and reverse 5′- CTCGCGCTGGCACAGATGCT -3′ for Flk-1 (reference sequence: NM_013062.1; [36]; forward 5′- TGGAGGAGGCTGACCGGCAA -3′ and reverse 5′- CTCTGTACCTCGGCAGGCCTGAAT -3′ for Caspase-3 (reference sequence: NM_012922.2; [37]; forward 5′- CAACACCTGAGCGGTTACCA -3′ and reverse 5′- AGAGGCAATGCGGTTCTGAT -3′ for COX-2 (reference sequence: NM_017232.3; [38]; forward 5′- ACGGCGTTTATCTCCACAAC -3′ and reverse 5′- CCGATGCACCTCTCAATG -3′ for PGF2α receptor (reference sequence: NM_013115.1; [39] and forward 5′- TCCACCCGCGAGTACAACCTTCTT -3′ and reverse 5′- CGACGAGCGCAGCGATATCGT -3′ for β-actin (reference sequence: NM_031144.3; [40].

### Statistical analysis

Significant differences in the mean values between the experimental groups were determined by a one-way analysis of variance (ANOVA). A Student Newman Keuls Test was used to compare the groups, and differences were considered significant if p < 0.05.

## Results

### Induction of thyroid dysfunction

**Figure 1:**
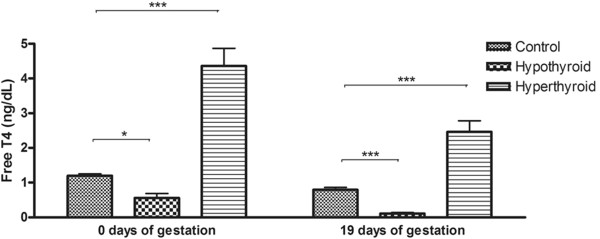
**Free T4 levels (means ± SD) in the plasma of pregnant rats of the control, hypothyroid and hyperthyroid groups at 0 and 19 days of gestation.** (*P < 0.05).

The induction of hypo-and hyperthyroidism during the entire period of the pregnancy was confirmed by serum free T4 at days 0 and 19 of gestation. The rats treated with PTU displayed free T4 levels lower than those of the control group (P < 0.05) (Figure 1). The thyroxine-treated rats exhibited higher free T4 levels compared to control animals (P < 0.05) (Figure 1).

### Proliferative activity

**Figure 2:**
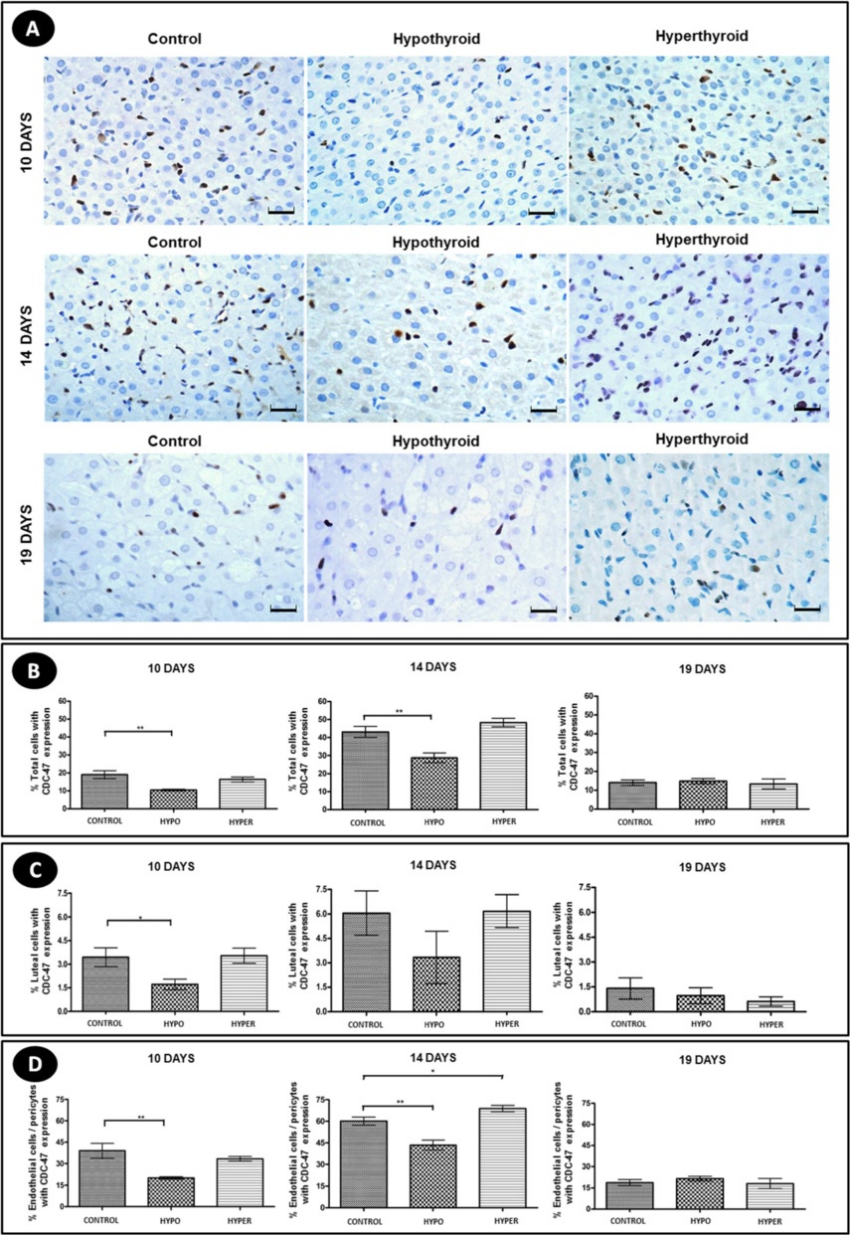
**Proliferative activity in the corpus luteum of pregnant rats of the control, hypothyroid and hyperthyroid groups at 10, 14 and 19 days of gestation. A)** Immunohistochemical images of CDC-47 expression. Marked reduction of the number of cells with CDC-47 expression in the corpus luteum of the hypothyroid group compared to the control group at 10 and 14 days of gestation (Streptavidin-biotin-peroxidase, Harris hematoxylin, scale bar = 12 μm). **B-D)** Percentage of cells with CDC-47 (means ± SD) expression in histological sections of the corpus luteum. (*P < 0.05; **P < 0.01; ***P < 0.001).

Regardless of the experimental group, the CDC-47 immunohistochemical expression in the corpus luteum was more intense at 14 days of gestation compared to other gestational periods (Figure 2).At 10 days of gestation, the CL of the hypothyroid animals showed a reduction in the total percentage of cells expressing CDC-47 compared to control rats in both the luteal cells and in endothelial cells and pericytes (P < 0.05) (Figure 2). The reduction of CDC-47 expression in the endothelial cells and pericytes in the CL of the hypothyroid animals persisted at 14 days of gestation; however, at 19 days there was no difference compared to the control group (p > 0.05) (Figure 2).The hyperthyroid group was different from hypothyroid rats at 14 days of gestation, showing an increase of CDC-47 expression in endothelial cells and pericytes compared to the control group (P < 0.05) (Figure 2). At 19 days of gestation, the group with hyperthyroidism showed no difference in their CDC-47 expression in the CL compared to the control group (p > 0.05) (Figure 2).

### Apoptotic activity (TUNEL and Caspase-3)

**Figure 3:**
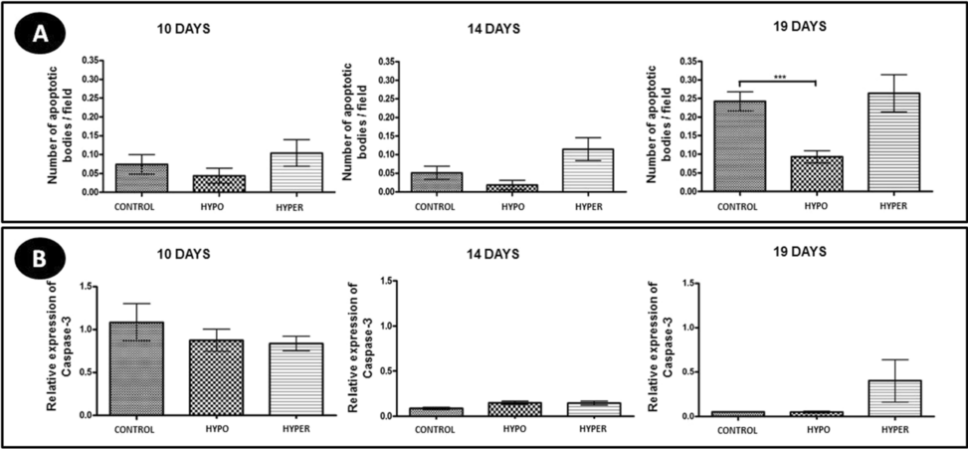
**Number of apoptotic cells/field and caspase 3 expression in the corpus luteum of pregnant rats of the control, hypothyroid and hyperthyroid groups at 10, 14 and 19 days of gestation. A)** Number of apoptotic cells/field (mean ± SD). **B)** Relative expression of gene transcripts for caspase 3 (mean ± SD). (*P < 0.05;**P < 0.01; ***P < 0.001).

The number of apoptotic cells in the CL of hypothyroid animals was lower compared to control group at 19 days of gestation (P < 0.05), while the hyperthyroid animals showed no differences compared to control rats (p > 0.05) (Figure 3A). The gene expression of caspase-3 did not differ significantly between the experimental groups in the three gestational periods (Figure 3B).

### Immunohistochemical expression of COX-2

**Figure 4:**
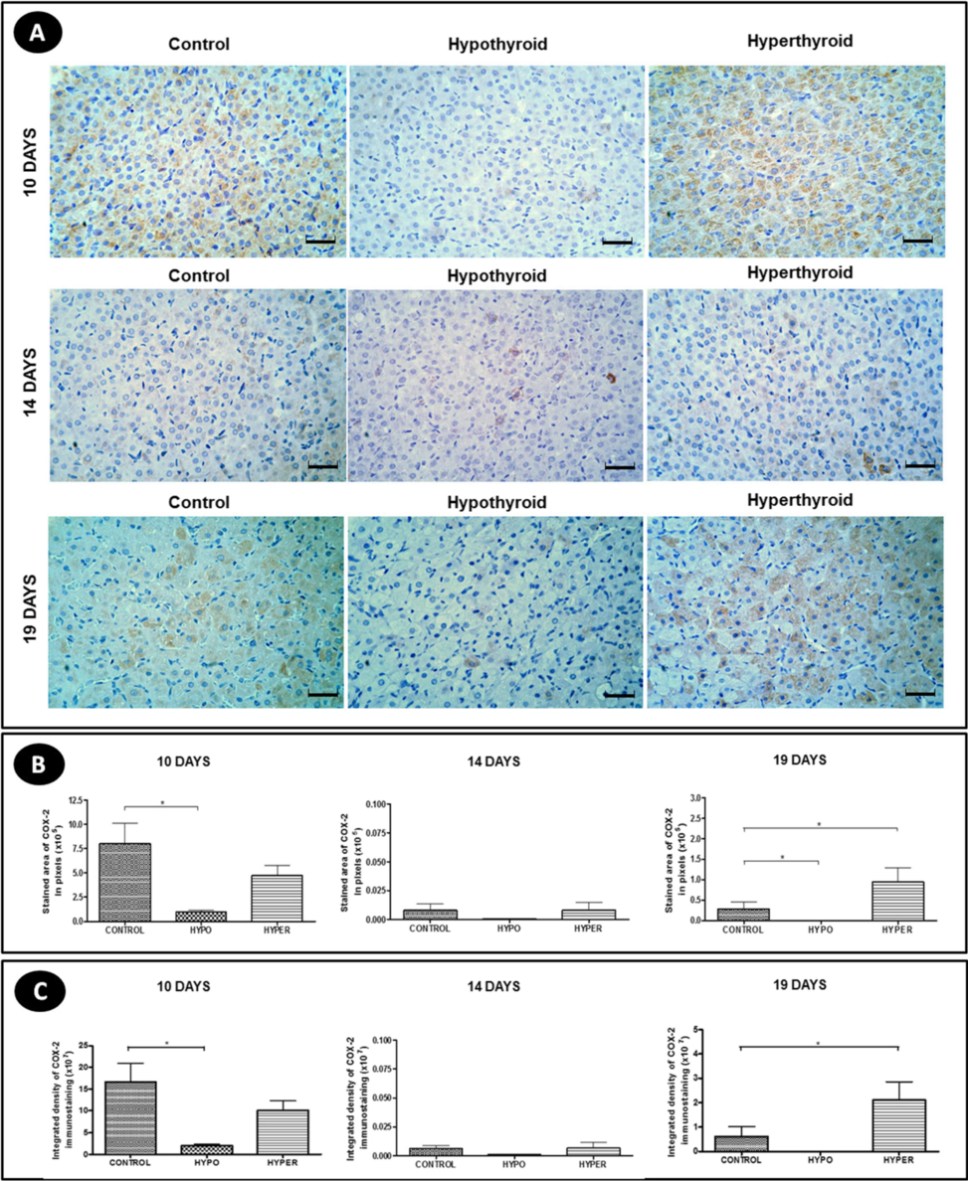
**COX-2 expression in the corpus luteum of pregnant rats of the control, hypothyroid and hyperthyroid groups at 10, 14 and 19 days of gestation. A)** Immunohistochemical images of COX-2 expression (Streptavidin-biotin-peroxidase, Harris hematoxylin, scale bar = 12 μm). **B** and **C)** Reduction of the area and intensity of COX-2 expression in the corpus luteum of hypothyroid group compared to the control group at 10 and 19 days of gestation. The hyperthyroid group had an increase of the area and intensity of COX-2 expression in the corpus luteum compared to the control group at 19 days of gestation.

Regardless of the experimental group, the COX-2 immunohistochemical expression in the luteal cells was cytoplasmic, with a more intense expression at 10 days of gestation compared to other gestational periods (Figure 4A).At 10 and 19 days of gestation, the hypothyroid animals showed a reduction of the area of COX-2 expression in the CL compared to the control group as well a reduction in the intensity of COX-2 expression at 10 days of gestation (P < 0.05). However, there was no difference at 14 days of gestation compared to the control group (P > 0.05) (Figure 4B and 4C). In contrast, hyperthyroidism at 19 days of gestation showed an increase of the area and intensity of COX-2 immunohistochemical expression in the CL compared to the control group (P < 0.05), while at 10 and 14 days of gestation no significant differences were noted (p > 0.05) (Figure 4B and 4C).

### Relative expression of gene transcripts to COX-2 and PGF2α receptor

**Figure 5:**
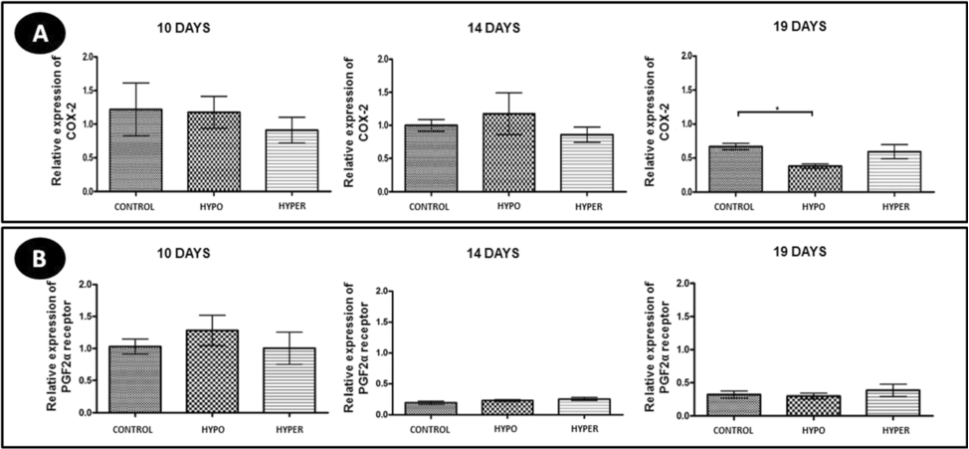
**COX-2 and PGF2α receptor expression in the corpus luteum of pregnant rats of the control, hypothyroid and hyperthyroid groups at 10, 14 and 19 days of gestation. A)** Relative expression of gene transcripts for COX-2 (mean ± SD). **B)** Relative expression of gene transcripts for PGF2α receptor (mean ± SD). (*P < 0.05).

Similar to the results of immunohistochemical analysis, the gene expression of COX-2 in hypothyroid animals was lower compared to the control group at 19 days of gestation (P < 0.05), while at 10 and 14 days of gestation no significant differences were noted (p > 0.05) (Figure 5A). The hyperthyroid group showed no significant difference compared to controls over the three gestational periods (p > 0.05) (Figure 5A). The gene expression of PGF2α receptor was not significantly different from that of the experimental groups in any of the gestational periods (p > 0.05) (Figure 5B).

### VEGF and Flk-1 expression

**Figure 6:**
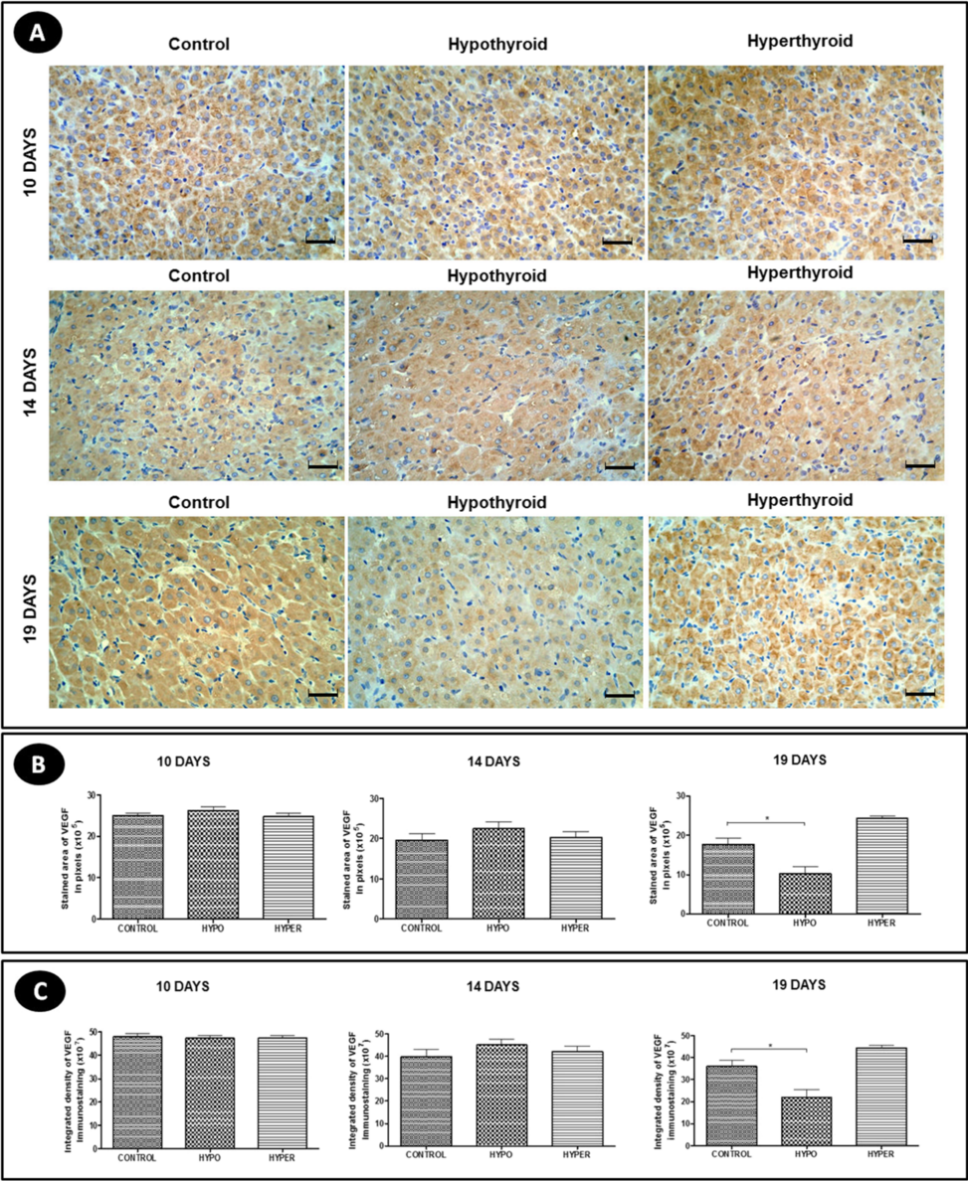
**VEGF expression in the corpus luteum of pregnant rats of the control, hypothyroid and hyperthyroid groups at 10, 14 and 19 days of gestation. A)** Immunohistochemical images of VEGF expression (Streptavidin-biotin-peroxidase, Harris hematoxylin, scale bar = 12 μm). **B** and **C)** Reduction of the area and intensity of VEGF expression in the corpus luteum of the hypothyroid group compared to the control group at 19 days of gestation.

**Figure 7:**
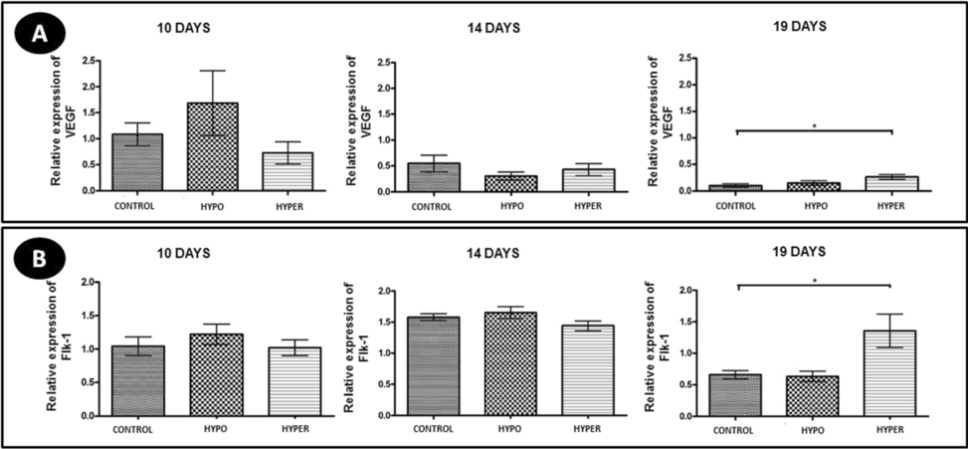
**VEGF and Flk-1 expression in the corpus luteum of pregnant rats of the control, hypothyroid and hyperthyroid groups at 10, 14 and 19 days of gestation. A)** Relative expression of gene transcripts for VEGF (mean ± SD). **B)** Relative expression of gene transcripts for Flk-1 (mean ± SD). (*P < 0.05).

**Figure 8:**
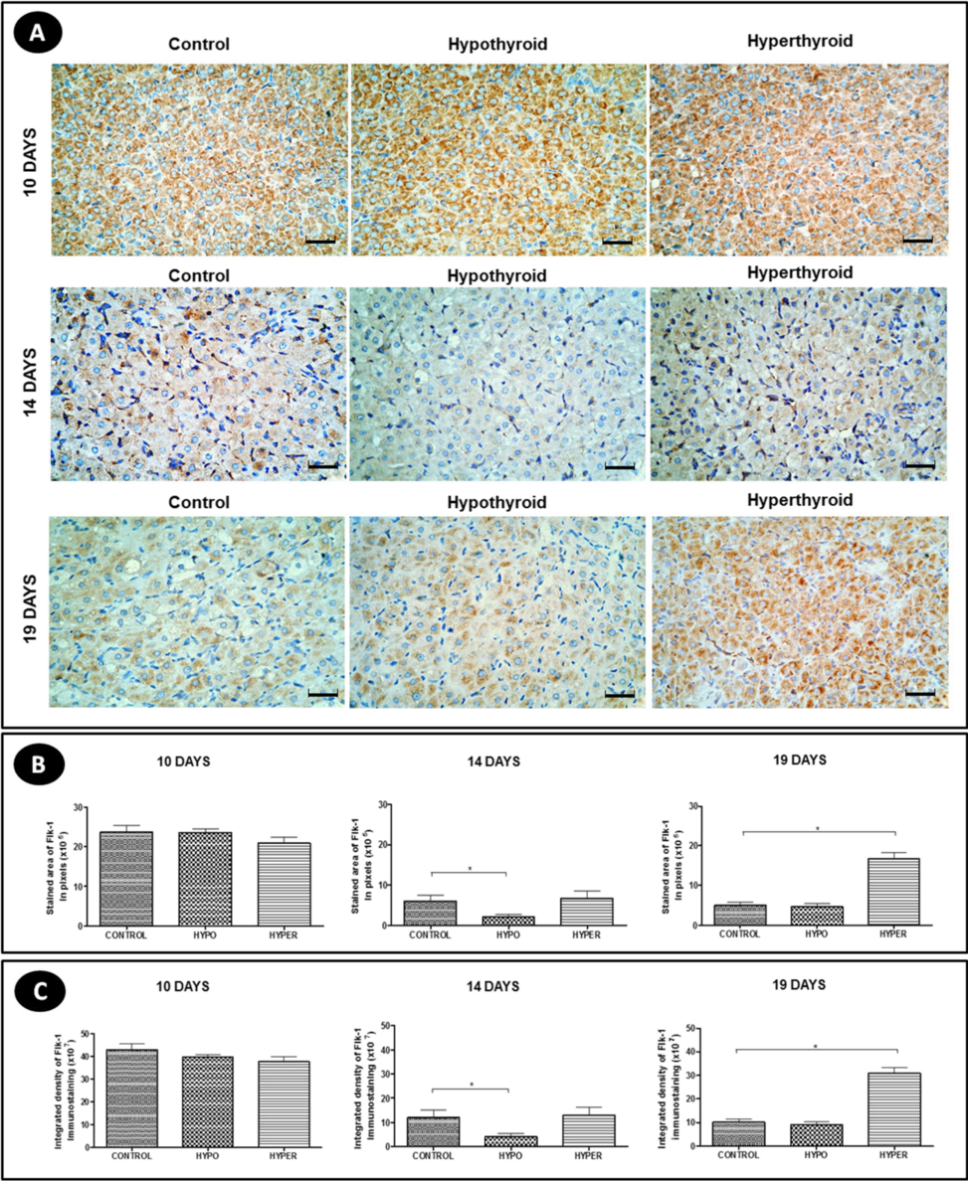
**Flk-1 expression in the corpus luteum of pregnant rats of the control, hypothyroid and hyperthyroid groups at 10, 14 and 19 days of gestation. A)** Immunohistochemical images of Flk-1 expression (Streptavidin-biotin-peroxidase, Harris hematoxylin, scale bar = 12 μm). **B** and **C)** Reduction of the area and intensity of Flk-1 expression in the corpus luteum of the hypothyroid group compared to the control group at 14 days of gestation. Increase of the area and intensity of Flk-1 expression in the corpus luteum of hyperthyroid group compared to the control group at 19 days of gestation.

Regardless of the experimental group and pregnancy period, VEGF expression in CL was evident mainly in the luteal cells, as its expression was cytoplasmic in all luteal cells (Figure 6A).Hypothyroidism reduced the area and intensity of VEGF immunohistochemical expression at 19 days of gestation compared to controls (P < 0.05) (Figure 6B and 6C), but no significant difference in the gene expression of VEGF in any period of pregnancy was found (p > 0.05) (Figure 7A). The hyperthyroid animals, differing from the hypothyroid group, showed no significant difference in the area and intensity of VEGF expression compared to control animals (p > 0.05) (Figure 6B and 6C). However, at 19 days of gestation, the hyperthyroid group showed an increase in the gene expression of VEGF compared to the control group (P < 0.05) (Figure 7A).The expression of the VEGF receptor Flk-1 occurred both in the luteal cells and in the endothelial cells and pericytes in the three experimental groups, with strong expressions of this receptor noted in luteal cells at 10 days of gestation and in endothelial cells and pericytes at 14 days of gestation (Figure 8A).At 14 days of gestation, hypothyroidism reduced the area and intensity of Flk-1 expression compared to control animals (P < 0.05), with no significant difference in any of the other periods of pregnancy (p > 0.05) (Figure 8B and 8C). The hyperthyroid animals, differing from the hypothyroid group, showed an increase in the area and intensity of Flk-1 expression compared to the control group at 19 days of gestation (p > 0.05) (Figure 8B and 8C). Similar to the results of the immunohistochemical expression, the hyperthyroid animals also demonstrated an increase in the gene expression of Flk-1 compared to control animals at 19 days of gestation (P < 0.05); however, they showed no significant difference in any of the other periods of pregnancy (p > 0.05) (Figure 7B).

## Discussion

The effects of hypo-and hyperthyroidism on proliferation, apoptosis and expression of angiogenic factors and COX-2 in the corpus luteum among the pregnant rats in this study were distinct.

Hypothyroidism significantly reduced the proliferation rate in the corpus luteum, both of luteal cells and of endothelial cells and pericytes. This result demonstrates that thyroid hypofunction affects not only the mitotic activity of the luteal cells of pregnant rats but also of the vascular cells. On the other hand, the corpus luteum of hyperthyroid animals showed an increased proliferative activity of the endothelial cells and pericytes at 14 days of gestation, suggesting the role of thyroid hormones on luteal angiogenesis. This is in agreement with Macchiarelli et al. [41] who observed that thyroid hormones stimulate the luteal angiogenesis. Our result is important because deficiency in luteal angiogenesis has been reported to substantially contribute to subfertility [17], and hypothyroidism in women cause miscarriage and stillbirth [11,12].

During luteinization, the cells of the corpus luteum must stop their proliferative activity to participate in steroidogenesis [8]. The phenotype of a differentiated luteal cell depends on the specific combination of genes encoding key regulatory proteins, such as receptors, transcription factors and signaling proteins. This reprogramming of follicular cells into luteal cells is irreversible and requires the cell to exit from the cell cycle [8]. For the luteal cells to exit from the cell cycle they must express p27^kip1^ that is a Cdk inhibitor. Cdk controls the G1 phase of the cell cycle together with cyclins [8]. It is likely that this reduction in the proliferative activity of the luteal cells found in the corpus luteum of hypothyroid animals is related to the higher plasma levels of progesterone presented by these animals [27], as a luteal cell’s capacity for progesterone synthesis is directly related to its degree of differentiation [8].

The reduction in the proliferation of endothelial cells and pericytes observed in hypothyroid pregnant rats may be a result of the decrease in COX-2 or Flk-1 expression. COX-2 is involved in PGE2 synthesis [21], which in turn stimulates angiogenesis [23]. On the other hand, signaling between VEGF and its receptor, Flk-1, is the main route [42] through which VEGF stimulates the proliferation of endothelial cells [19]. Kashida et al. [43] demonstrated that VEGF induces angiogenesis in the corpus luteum and is involved in the increase in size of the corpus luteum during mid-pregnancy, being that the VEGF expression is regulated by TNFα [44]. TNFα is also involved in the corpus luteum regression and inhibits progesterone production in vitro by the luteal cells [45,46]. However, further studies are needed regarding the relation between TNFα and thyroid dysfunction on the corpus luteum. The hypothyroid pregnant rats in this study also showed a significant reduction in their expression of placental lactogen-1 (PL-1) (unpublished data), which could represent an additional mechanism to explain the reduction of the proliferative activity of the endothelial cells of the corpus luteum because the PL-1 is also important in maintaining luteal angiogenesis in rats [47].

Regarding apoptosis, hypothyroidism reduced the number of apoptotic cells in the corpus luteum at 19 days of gestation. This is most likely associated with the delay in COX-2 expression presented by these animals compared to the control group. The lower COX-2 expression found in the corpus luteum of hypothyroid animals can cause a decrease in the formation of PGF2α [21]. This result may explain the prolonged luteal phase in hypothyroid pregnant rats and, consequently, the delay in the fall of circulating progesterone and delivery experienced with these animals [28,48]. PGF2α is crucial for the increase in the luteal expression of the 20α-hydroxysteroid dehydrogenase (20αHSD) at the end of pregnancy, and that gives the signal for parturition in rodent [49]. 20αHSD converts luteal progesterone into its inactive metabolite (20α-dihydroprogestagen) [21]. Hapon et al. [48] observed that the delay in progesterone decline and parturition in rats with hypothyroidism is caused by a decrease in luteolytic factors, mainly luteal PGF2α, addition to an increase of luteotrophic factors, such as PGE2 and prolactin.

Higher plasma levels of progesterone in hypothyroid rats [27] may also favor the reduction of the number of apoptotic cells observed in corpora lutea of these animals, since progesterone suppresses the activity of caspase-3 [50,51], apoptosis and degeneration [52] in the corpus luteum. However, hypothyroid animals there were no difference in the gene expression of caspase-3. Corpus luteum obtained from caspase-3 null mice show attenuated rates of apoptosis and delay in the process of involution [53], but the corpus luteum of them involutes. This shows that caspase-3 is not the sole factor leading to cell death in this gland [8,53]. Recently, various forms of programmed cell death (apoptosis, necrosis and autophagy) have been suggested as potentially being triggered during the regression of the corpus luteum; programmed cell death depends of the animal species, the physiological or pathological conditions assessed and/or the nature of the luteolytic stimulus [8,54]. In addition, further studies are needed to assess in greater detail the process of apoptosis in the corpus luteum of animals with thyroid dysfunction, such as via Fas/FasL, to provide a better understanding of the process of luteal regression that occurs in the corpus luteum of these animals.

Hyperthyroid animals showed an increase in mRNA expression for VEGF and Flk-1 at 19 days of gestation and an increase in Flk-1 immunohistochemical expression, which was distinct from the results of hypothyroid animals that experienced a reduction of VEGF immunohistochemical expression. Silva et al. [33] observed that, during the diestrus of non-pregnant rats, there is an increase in the intensity of Flk-1 and VEGF expression in the luteal cells of the corpus luteum of previous cycles and in regression compared to the newly formed corpus luteum. Furthermore, the process of luteal regression occurs in a hypoxic environment, as low oxygen tension stimulates VEGF expression and/or the expression of its receptor through the expression of hypoxia-inducible factor-1 (HIF-1) [55]. Wada et al. [56] also observed a reduction of the luteal blood flow at the end of the pregnancy period in mice before any of the structural changes of luteal regression. As hyperthyroid animals undergo an early luteal regression [29], one hypothesis is that a hypoxic environment has already been developed in the corpus luteum of these animals at 19 days of gestation, justifying the higher expression of VEGF and Flk-1 in relation to control animals.

Regarding hypothyroid animals, a lowered VEGF immunohistochemical expression at 19 days of gestation could compromise luteal function, as VEGF influences the production and release of progesterone by the corpus luteum [42] and controls its vascular permeability [56]. More research is needed to assess how hypothyroidism affects luteal function to the detriment of the alterations observed in the VEGF expression because this thyroid dysfunction results in abortion in the final third of pregnancy and stillbirth [11].

## Conclusion

Experimental hypothyroidism reduces the proliferation, apoptosis and expression of angiogenic factors and COX-2in the corpus luteum of pregnant rats. In contrast, experimental hyperthyroidism increases the expression of angiogenic factors and COX-2 and the proliferative activity in the corpus luteum of pregnant rats.

## Abbreviations

CDC-47: Cell division control protein 47; VEGF: Vascular endothelial growth factor; Flk-1: Fetal Liver Kinase 1; COX-2: Cyclooxygenase 2; PGF2α: Prostaglandin F2alpha; PTU: Propylthiouracil; CL: Corpus luteum; Cdk: Cyclin-dependent kinase; TNFα: Tumor necrosis factor alpha; PL-1: Placental lactogen 1; 20αHSD: 20α-hydroxysteroid dehydrogenase; PGE2: Prostaglandin E2; HIF-1: Hypoxia-inducible factor-1.

## Competing interests

The authors declare that they have no competing financial or non-financial competing interests.

## Authors’ contributions

JS and RS conceived and designed the study. JS performed the experiments of immunohistochemistry and molecular biology and wrote the manuscript. NO and RS contributed to the writing and to the critical reading of the paper. All authors read and approved the final manuscript.
